# Minimal self-consciousness and the flying man argument

**DOI:** 10.3389/fpsyg.2023.1296656

**Published:** 2023-12-14

**Authors:** Shaun Gallagher

**Affiliations:** ^1^Department of Philosophy, The University of Memphis, Memphis, TN, United States; ^2^School of Liberal Arts, University of Wollongong, Wollongong, NSW, Australia

**Keywords:** flying man, Avicenna, minimal self-consciousness, sensory input, interoception

## Abstract

The concept of minimal self-consciousness or “minimal self” is equivalent to a very basic form of first-person, pre-reflective self-awareness, which includes bodily self-awareness, and is related to phenomenal experience (qualia) and sentience. This phenomenological concept plays a role in characterizations of the senses of ownership and agency; in recent debates about Buddhist conceptions of the no-self; in explanations of illusions such as the Rubber Hand Illusion; as well as in characterizations of schizophrenia as a self-disorder. Despite its relevance to these complex investigations, a number of theorists have recently pointed out that the concept is not well defined. In order to provide some clarification about the notion of minimal self and how it relates to bodily and sensory processes this paper reaches back to the ideas expressed in a famous medieval thought experiment proposed in the 11th century: Avicenna’s Flying Man argument. The paper then provides a review of some of the contemporary debates about the minimal self, pointing especially to questions about the role of bodily and social processes.

## Introduction

The phrase “minimal self,” as used in this paper, and as it has been used in both cognitive science and phenomenological philosophy of mind, is equivalent to a very basic form of first-person, pre-reflective self-consciousness, which includes bodily self-awareness. This concept plays a role in phenomenological characterizations of the sense of ownership, and the sense of agency (Gallagher, 2000a; Zahavi, 2017); in recent debates about Buddhist conceptions of the no-self (Albahari, 2011; Siderits et al., 2011); in explanations of illusions such as the Rubber Hand Illusion (Limanowski, 2014; Georgie et al., 2019); as well as in characterizations of schizophrenia as a self-disorder (Nelson et al., 2014). Despite its relevance to these complex investigations, Kim and Effken (2022, 15), have recently pointed out that “there are no clear criteria to define the minimal self except for some vague intuitive feeling of ‘a basic, immediate, or primitive ‘something’ that we are willing to call a self”’ (citing Gallagher, 2000a). Likewise, Lang and Viertbauer (2022) outline a plethora of views on pre-reflective self-awareness, and conclude that given this range of interpretations it “is not surprising that there is not only controversy about what is meant by pre-reflective self-consciousness, but moreover whether pre-reflective self-consciousness exists at all …”

These controversies relate to other concepts relevant to understanding consciousness, namely, phenomenal experience (qualia) and sentience, especially as the latter is defined by Nicholas Humphrey, who reaches back to the early seventeenth century to find its original meaning: what sensations feel like to the subject who responds to sensory stimuli (2022, 1). In order to provide some clarification about the notion of minimal self and how it relates to bodily and sensory processes I will reach back a bit further in the history of these ideas to a famous medieval thought experiment proposed in the 11th century: Avicenna’s Flying Man argument. I’ll then review some of the contemporary debates about this concept.

Let me preface the following with a methodological proviso. Like empirical experiments that require the introduction of controls, and like toy models that introduce unrealistic simplifications, thought experiments are also limited in terms of the kinds of results we can attain through their use. In all of these approaches one ends up with some degree of abstraction from the phenomenon that one is attempting to explain. In the following sections I’ll often be discussing abstractions. I’ll argue, however, that they are insightful abstractions that can provide some direction for further thinking. In that respect the strategy is to point out how they are abstractions and to point to a trajectory that could result in less abstract insights, even if in this paper we don’t have the space to pursue these trajectories. In my view, all such trajectories lead toward more embodied and enactive approaches to issues concerning pre-reflective self-awareness. It is specifically the limitations of the more abstract, less embodied views that point us in the right direction.

## The flying man

Although philosophers often use thought experiments rather than empirical experiments to further an argument, many philosophers (not only today, but also in the past) engage or have engaged with empirical studies, and Avicenna is no exception to this. As both a physician and a philosopher he conducted empirical medical research and, in the 11th century, published a work entitled *The Canon of Medicine*, which came to be used in western universities until the 16th century. The third volume of this work includes chapters on spinal cord injury (Ghaffari et al., 2022). It’s notable that considerations of spinal cord injury have more recently played a strategic role in addressing a question that is roughly similar to the one that Avicenna addresses in his thought experiment on the Flying Man. For example, in behavioral and neuroscientific studies of spinal cord injury Moro et al. (2022), ask whether and to what degree the body’s sensory and motor processes, or lack thereof, contribute to or constrain cognition. They develop a positive answer showing how, even in severe cases of body-brain disconnection, deafferentation and de-efferentation, embodied processes continue to play a role in modulating a broad range of cognitive capacities, including spatial perception, motor imagery, the discrimination of biological motion, affordance perception, and so forth. In contrast, Avicenna’s seemingly negative answer in the Flying Man argument focuses on just one narrow question about self-awareness. At the end of the first chapter of his treatment of soul in the *Psychology*, Avicenna (Ibn Sina) (1959) argued that a newly created man would be self-aware even if he were floating in a void with all his senses disabled.^1^ The conclusions to be drawn are that the self that one is aware of is not bodily, and self-awareness is not an awareness by means of the senses.

Avicenna presents several versions of the argument. The most extensive one is this:

One of us must suppose that he is created all at once, and created as perfect, but with his sight prevented from seeing anything external [to him]. He is created hovering in the air, or in a void, in such a way that the air does not buffet him so that he would have to feel it. His limbs are separated so that they do not meet or contact one another. He must then reflect as to whether he will affirm the existence of his self [*dhaāt*]. He will not hesitate to affirm himself to exist. He will not, however, affirm things exterior to his members nor the hidden things of his interiors nor his soul nor his brain nor anything else extrinsic. He will affirm himself to exist though he will not affirm the length or the width or the thickness of himself. If in this situation he were able to imagine a hand or another limb, he would not imagine it as a part of himself, nor as a condition for his self…. As to the self whose existence he affirms, it is specific for it that it is identical to him and distinct from his body or his limbs, which he has not affirmed. Thus the alert person has a way to be advised concerning the existence of the soul [or self] as something distinct from the body, or rather distinct from body, and [a way] by which he may understand it and be aware of it. (1959, 15–16; trans. modified from Adamson and Benevich (2018), 148–149).

There is some scholarly dispute about the meaning of the word *dhaāt* (self or essence).^2^ For purposes of this paper, I set aside the ontological-terminological issues in order to focus just on the phenomenology—and for that purpose, I translate *dhaāt* as “self,” following a precedent set by Marmura (1986, 383) who argued, “The primary concern [of the argument] is psychology, not metaphysics.” This allows us to focus on a point that scholars generally agree on, namely that Avicenna designed the flying man to argue that being aware of oneself is independent of any visual, tactile or proprioceptive awareness of one’s body or any further content of experience (Avicenna (Ibn Sina), 1959, 225). Thus, he argues that if you are completely unaware of your body and your physical circumstances, you would be unaware of everything except the “fixedness” of your individual existence (1959, 225). This is a form of self-awareness, Avicenna argues, that is a constituent of the self, and “belongs to it always and in actuality”—a form of natural knowledge that does not depend on contact with another human.

Adamson and Benevich (2018) note that Avicenna elsewhere claims, “we are constantly aware of ourselves, even when asleep,” which they interpret to mean that this is a form of tacit self-awareness.^3^ Avicenna himself suggests that most of the time we are not “alert” to this awareness, i.e., that we do not have reflective knowledge of it (1959, 226–227). Kaukua (2020, 13–14) interprets this as follows: “most of us have no experience of being aware of nothing but ourselves, given that in the normal circumstances, we are aware of ourselves as embodied agents and subjects of cognition, constantly immersed in our mutual engagement with the world around us. The [flying man] argument is designed to show that self-awareness would remain even if these features normally associated with it were bracketed. It points at something, ourselves, the existence of which we assert without asserting the existence of anybody.” As we’ll see, however, and as frequently noted (see Black, 2008) this doesn’t mean that the self is disembodied. Indeed, self-awareness does not tell us *what* the self is. “Surely, one may be aware of the existence of something, including oneself, without knowing what that thing is, and it is precisely such an awareness of existence that the flying man has.” (Kaukua, 2020, 11). We might argue, however, that self-awareness does tell us one thing about what the self is—it is something that, at a minimum, is capable of self-awareness. And Avicenna says something like this: “[the soul or self’s] awareness of itself is *by nature*, this being a constituent of it and hence belongs to it always and in actuality” (cited in Marmura, 1986, 386; emphasis added).

Self-awareness is a form of what Avicenna calls natural knowledge.^4^ He uses the following example to define natural knowledge. He suggests that if a person is created fully mature and rational, having, however, had no contact with other humans and human institutions, and is confronted with a commonly accepted moral dictum and a self-evident logical truth, he will be able to doubt the first, but not the second (Avicenna (Ibn Sina), 1892, 119; discussed in Marmura, 1986). Although Avicenna recognizes the importance of intersubjective interaction, specifically in the ethical context, he argues that natural knowledge is not something we learn from anyone else. This is the kind of knowledge had by the flying man, i.e., a person born fully mature and rational but having had no human contact. Avicenna thus holds that the self has natural, constant knowledge of itself.

## The minimal self

Much of the contemporary discussion about the minimal self was motivated by Strawson’s (1997) essay on the self.^5^ There he indicated the methodological primacy of phenomenology over ontology and simply asked what was the most minimal experience of self that we could have. He considers the answer to this to be very basic, and “situated below any level of plausible cultural variation” (§3). His answer is that this basic self is a “mental self.” Although he is a philosophical materialist, and believes that we are wholly material things, the characterization of the self as mental is an answer to the strictly phenomenological question of what we experience.

With respect to this minimal mental self Strawson excludes diachronicity, agency, and personality. For example, he writes:

It seems plain that … experience of the self does not necessarily involve experience of it as something that has a personality. Most people have at some time, and, however, temporarily, experienced themselves as a kind of bare locus of consciousness—not just as detached, but as void of personality, stripped of particularity of character, a mere (cognitive) point of view. Some have experienced it for long periods of time. It may be the result of exhaustion or solitude, abstract thought or a hot bath. It is also a common feature of severe depression, in which one may experience “depersonalization.” This is a very accurate term, in my experience and in that of others I have talked to. (1997, 420).

Diachronicity is set aside based on Strawson’s own phenomenology (now relatively famous in philosophical circles), that he experiences at best a 3-s-long self, and is not inclined to narrative extensions (also see Strawson, 2004). He states: “I believe the Buddhists have the truth when they deny the existence of a persisting mental self, in the human case, and nearly all of those who want there to be a self-want there to be a persisting self” (1997, 427).

Strawson also excludes the sense of agency, although he does not provide an argument for this exclusion. We can suppose that a sense of agency only comes along as we are engaged in some action, and when we are not, we don’t have a sense of agency, so it can’t be essential. We could add Avicenna’s view on this. He raises the question of whether self-knowledge is mediated through one’s action. This, he argues, is not the case because, “the supposition” of the flying man argument excludes any action. Moreover action is either general or specific. General action does not lead to the knowledge of the particular self. The action would have to be particular; for example, my own individual act. But when I state that I am performing an act, the “I” is prior to my act. My act presupposes the existence of my-self; otherwise I would not refer to it as *my* act (see Black, 2008, and similar points made in regard to object perception in Avicenna (Ibn Sina) (1973), 161).

More positively Strawson defines the “minimal case,” or “the minimal form of self-experience” as a momentary (single) mental subject of experience.^6^ He refers to this as mental or M-experience and asks whether this is clearly a case of *self*-experience, to which he answers “yes,” but notes that we are one step away from the Buddhist idea of non-self.

[I]t is not clear that the minimal case of Self-experience is *ipso facto* the minimal case of M-experience. I suspect that the minimal case of M-experience may be some kind of “pure consciousness” experience, of the kind discussed by Buddhists and others, that no longer involves anything that can usefully be called “Self-experience” at all (1999, 118).

He calls this the “meditative rider” to his positive claims, namely that genuine “M-experience” need not involve an experience of self. If we stay with the concept of minimal self-experience, however, Strawson’s position is close to the standard phenomenological view, namely, that self-experience is not an experience of some object. In this regard he quotes the phenomenologist Louis Sass, who, in turn, references William James: the self “is not, in fact, experienced as an entity in the focus of our awareness, but, rather, as a kind of medium of awareness, source of activity, or general directedness toward the world” (Sass, 1998, 562). Strawson then translates this into the terminology preferred by analytic philosophy: although the self is experienced as a thing of some sort, this “does not require experience of self that is experience (as) of ‘an entity in the focus of awareness”’ (1999, 115).^7^ Strawson also quotes the commentary by Zahavi and Parnas (1998), which refers to “the basic self-awareness of an experience,” as “an immediate and intrinsic self-acquaintance which is characterized by being completely irrelational” [Zahavi and Parnas (1998), p. 696]. “Irrelational” here means it does not have a subject-object structure, but rather is solely the subject with the structure of pre-reflective self-awareness.^8^

## The phenomenology of minimal self-awareness

This phenomenological conception of the minimal self, however, is not an entirely settled issue, and contemporary debates focus on several questions.

1. Is the minimal self *experiential*, or simply an abstract, formal notion?
2. Is the minimal self *embodied*?
3. Does the minimal self-involve *social* existence?

First, a quick answer to the first question is that it is experiential, specifically, as a structural feature of experience it is something that is experienced; it is more than simply a formal principle of the sort defined by Kant. Yet, it is in some regards an abstraction, since for the most part, in our everyday experience, it is never experienced solely in itself without other complications, which may involve embodiment and intersubjectivity. Here we may also start to see the limitations of Avicenna’s flying man in its attempt to abstract away from all sensory experience, including experience of the body.

Second, with regard to the question of embodiment, accounts of the minimal self often include references to proprioception, especially in relation to two features that are typically included in minimal self-awareness: the sense of ownership (or mineness, or “for-me-ness”) and the sense of agency. As we noted, however, the latter is present only when some form of action is involved. This is why Strawson excludes it as essential. To be clear, however, one may have a sense of agency not just for bodily action, in the sense that such action involves proprioception/kinesthesia, as well as efferent processes that may contribute to self-awareness. One may also have a sense of agency for thinking, imagining, remembering, etc. On most interpretations, of course, these cognitive processes involve some embodied aspects (embodied simulation, activations in motor areas, and perhaps even proprioceptive, affective and interoceptive processes), all of which seemingly tell us that we are the agent of such cognitive processes.

The sense of ownership or mineness, however, seems more basic. This was indicated by Avicenna when he suggested that my act (or agency) presupposes the existence of my-self; otherwise I would not refer to it as *my* act. Again, there are many studies that discuss the sense of bodily ownership—this includes, for example, experiments on the rubber-hand illusion (see Riemer et al., 2019; Ehrsson, 2020, 2023; for a recent review of this literature see Georgie et al., 2019).^9^ Moreover, on some interpretations, schizophrenic delusions of control and thought insertion represent cases in which the sense of agency is missing, but the sense of ownership remains [“It is my hand that did it, but I did not control it”; “I experience this thought as part of my stream of consciousness, but I did not think it” (Gallagher, 2004; Frith, 2015)]. In the latter respect the sense of ownership does not necessarily involve an explicit sense of body ownership. The more general claim, in regard to the minimal self, is that there is a very basic sense of mineness implicit in experience itself.

This just is the claim that when there is experience, there is a subject of experience, and phenomenologically this can be explained in terms of the temporal structure of consciousness. On Husserl’s account, the retentional structure of consciousness involves retaining in a continuous but fading manner the just-past experience, which allows me to say, for example, that I’ve been listening to a particular piece of music, without engaging in a full-blown act of recollection (Husserl, 1991). This immediate retention includes the sense that it is *my* ongoing experience—that I am the one who has been experiencing the music. The mineness of the experience is built into this structure, and I never have this immediate sense of experience for experience that is not my own.^10^

Concerning the question of embodiment, by design of Avicenna’s thought experiment, as in cases of sensory deprivation experiments, we exclude sensory input and bodily movement. With respect to proprioception, when you do not move for some time, your proprioceptive sense of where your limbs are located dissipates. The phenomenology is that in this circumstance, if I don’t move, without vision, I don’t know where my limb is because I can’t feel it. The subjective experience of position sense is not just less vivid or less precise; it has disappeared. To be sure, this is quite temporary. All I have to do is move my limb and proprioceptive awareness returns. Furthermore, however, we should note that in some experimental cases of anesthetic block of the sensory and motor nerves of the arm, the blocking of proprioception does not remove awareness of the limb; rather, a phantom arm is experienced (Melzack and Bromage, 1973), or one has contradictory experiences: an experience of the limb as missing and, at the same time, an illusory experience of the limb as enlarged or swollen or shrunken (Paqueron et al., 2003). One of the reviewers for this paper reports that in the case of a complete experimental ischemic block of one’s arm, which is non-visible behind a screen, proprioceptive awareness of the arm does not dissipate; one still has a sense of it somewhere behind the screen. However, when the screen is removed and the hand is visible, it no longer feels like one’s own arm because (due to proprioceptive drift) the visual input does not match its felt position. Accordingly, eliminating proprioception is not so straight forward, and for this reason in the flying man experiment we would need to stipulate, in line with Avicenna’s aims, that the person comes into existence in a condition of complete deafferentation (see Gallagher and Cole, 1995; Miall et al., 2021; Gallagher, 2022; for a discussion of empirical cases).

It may be even more difficult to get rid of interoceptive sensation, and in sensory deprivation experiments, these sensations are still operative. Indeed, sensory deprivation experiments suggest that interoception (of beating heart, respiration, hunger, pain, etc.) is enhanced when one removes extrasensory input (Feinstein et al., 2018). This is one important difference between sensory deprivation experiments and Avicenna’s flying man experiment, assuming that interoception is eliminated in the flying man. One might argue that just such interoceptive sensation, what James (1890) calls the “warmth and intimacy” of bodily sensations, or what Fuchs (2013) calls “the feeling of being alive”—a pre-reflective, bodily self-awareness that comprises the background of all intentional feeling—is part of what causally generates the basic sense of mineness for any of my experiences, and constitutively just is what I typically experience as my-self. The sense of body-ownership, then, could be said to depend on the formal temporal structure, the retention of my ongoing experience that, at a minimum, is interoceptive.^11^ Hence the importance for Avicenna of eliminating interoception, as well as proprioception and exteroception.^12^

Finally, some recent critical discussions of the minimal self ask whether the same can’t be said about intersubjective or social aspects of experience—that, like the body, they play some implicit role affecting minimal self-awareness. For example, Ratcliffe (2017) argues that the minimal self has to be re-conceptualized in interpersonal terms since the most basic sense of self is developmentally dependent upon other people. Zahavi (2017) responds to this point. He has no problems with Ratcliffe’s general claims about the importance of intersubjective dimensions, but he thinks they are irrelevant to the issue concerning the minimal self. He rejects the claim that this basic feature of consciousness “is interpersonally constituted such that young infants who had not yet engaged in sufficient interpersonal relations as well as all non-social organisms would lack phenomenal consciousness and minimal selfhood” (2017, 195). Zahavi’s view is consistent with Strawson’s view that even non-human (and non-social) animals can have a minimal self. Furthermore, Zahavi points out that there is a shift in Ratcliffe’s argument, such that in the end he does not deny a minimal self to infants and non-human animals, but rather would insist that social development brings along a transformation of the minimal self. The idea that social processes may transform the minimal self is an open question for Zahavi, but regardless of how one answers that, given the possibility of social transformation, the issue would no longer be the denial of a non-social minimal self, but a claim about how the minimal self may change in development. “Contrary to the (more) minimal self of an infant, the (less) minimal self of an adult is interpersonally constituted” (Zahavi, 2017, 195). In this case, the proposal by Ratcliffe is not incompatible with Zahavi’s notion of minimal self.

We saw that Avicenna defended a similar position, distinguishing natural knowledge from knowledge that we learn from others; minimal self-awareness is a form of natural knowledge that does not depend on others; one can see this in the case of the flying man, since not only is the flying man in a state of sensory deprivation, he is also in a state of intersubjective deprivation. One can think here of the communicative difficulties faced by subjects who are deaf-blind (Gallagher, 2017). Take away all of the other senses, and thereby all social interaction, would there not still remain a self-awareness? At least with respect to the question of the social, seemingly both Zahavi and Ratcliffe would agree with Avicenna’s positive answer.

Zahavi also responds in a very similar way to criticisms proposed by Ciaunica and Fotopoulou (2017; see Kyselo, 2016) who contend that the minimal self is intersubjectively constituted. He again accepts the idea that social factors may affect other aspects of the self, and may even transform minimal self-experience. If this were not the case, one would have to consider the minimal self as self-enclosed and not open to the world. Zahavi contends, in contrast, that “qua subject of intentional experience, [the minimal self] is inherently open to the world and others” (2017, 196). More to the point, the phenomenology of the self is not exhausted by the minimal self—there are other aspects of the self (for example, narrative features) that are shaped by intersubjective interactions.

Ciaunica and Fotopoulou (2017), however, present another argument that centers on interoception. They contend that interoception (the inner feelings of bodily arousal, wakefulness, wellness etc. that accompany physiological changes) is crucial for self-experience, and indeed for the self-other distinction. As indicated above, we can allow that implicit interoception may be an important contributor to minimal self-awareness, and this suggests that the minimal self is embodied, even if I do not, or if the flying man does not experience it as such. Ciaunica and Fotopoulou (2017), however, go beyond this point; they contend, interoceptive modalities depend upon and are changed by embodied interaction with others (see also Crucianelli and Ehrsson, 2023). One can think of physiological and affective regulation by others, not only in infancy, but throughout the life span. In this respect subjective “feeling states” are, at least in part, taken to be the result of such interactions, and do not pre-exist embodied social encounters. One response to this is to accept all but the last point, and rather insist that such interoceptive feeling states do pre-exist encounters with others, arguing, in agreement with Ratcliffe, that they can then undergo transformation in our embodied encounters with others.

This aligns with Zahavi’s response, that to read this late transformation into the initial natural phenomenal state would lead to the idea that the self is entirely socially constructed and does not exist outside of social relations—on that view, “human beings, who are deprived of the required social interaction and denied socially mediated attributions of self, would also lack me-ness, be self-less and without consciousness, and therefore remain ‘unconscious zombies”’ (2017, 198). This is the view he rejects.

Ciaunica and Fotopoulou (2017), however, do not accept the idea that social interaction is a late achievement. Ciaunica et al. (2021a,b) have argued, for example, that intersubjective interactions already exist between the fetus in the womb and the mother. One might think of this as a kind of primary intercorporeity (Merleau-Ponty, 2012). If one accepts this, then it may be that as consciousness initially emerges, the fetus is already affected by a kind of natural alterity or connection with the other that is somehow intrinsic to pre-reflective experience. There is certainly an argument to be made [and some empirical evidence (see Lymer, 2011)] about proprioception in fetal development providing a self/non-self-distinction. Whether that amounts to a self-*other* (intersubjective) distinction is an open question.

In this regard, I note that the flying man argument avoids or short-circuits this issue. The flying man “is created all at once, and created as perfect”—apparently not born of a mother, but created by God, where “perfect” seemingly does not depend on having sensory input or encountering others. Perhaps more relevant to the point made by Ciaunica et al. (2021a,b) the flying man is without sensory input (including, supposedly, proprioception, kinesthesis and interoception), especially the kind of sensory input that would provide some kind of access to or awareness of another person. Assuming that Avicenna would want to exclude interoceptive sensation, the flying man would offer resistance to the argument by Ciaunica et al. (2021a,b) since their argument depends on the multisensory basis of pre-reflective experience, specifically touch and interoception (Ciaunica and Crucianelli, 2019). Touch and the other exteroceptive senses are important because they are what allow access to others—touch especially in the case of the fetus. Without sensation of a sort that gives us access to others, would a minimal form of self-awareness with access only to my own embodied self-experience be possible? This is likely even more minimal than Zahavi would like, since he takes the minimal self to be inherently open to the world and others, which the flying man seemingly is not.

## Conclusion: the super flying man

What the flying man argument shows is that the minimal self is something genuinely experiential, but at the same time an abstraction. An abstraction because to arrive at the concept of the minimal self one has to set up a thought experiment where you remove everything that contextualizes human experience, including almost all embodied sensory experience. “Almost,” because, even if one manages to eliminate proprioception and the vestibular sense, it remains a challenge to eliminate interoception. As noted, in sensory deprivation experiments, interoception may even be enhanced when one removes extrasensory input. The elimination of interoception is, of course, an empirical issue. Although the anterior insula has been identified as integrating “all subjective feelings from the body and feelings of emotion” (Craig, 2002, 655), more recent studies demonstrate that it’s much more complicated. Body ownership and multisensory integration involves a complex network that includes frontal and parietal association cortex, such as the premotor cortex and the posterior parietal cortex (Ehrsson et al., 2004; Gentile et al., 2013; Limanowski and Blankenburg, 2016; Guterstam et al., 2019; Chancel et al., 2022; Abdulkarim et al., 2023). Furthermore, there is an additional source of interoceptive sensations – the skin and its somatosensory afferent projections (Khalsa et al., 2009; Rudrauf et al., 2009; Crucianelli and Ehrsson, 2023).

Since this is a thought experiment, we can ideally lesion the projections from skin to somatosensory areas of the brain, as well as knock out any areas responsible for multisensory integration and body ownership and then assume that such operations would entirely eliminate interoception. At this point we would have to leave aside the question of whether we could do something like this and not affect any other of the person’s capacities, so that he would be “perfect” (except for sensation), as stipulated by Avicenna.^13^ This indeed would be a super flying man, with no internal or external sensations.^14^ Would he still have a minimal self-awareness—a super-minimal self-awareness?

To answer this question one needs to distinguish between the content and structure of phenomenal consciousness. On Avicenna’s view sensory content is not the determining factor for minimal self-awareness (see Black, 2008, 68–69). Appealing to the flying man argument he argues that self-awareness is completely autonomous and independent of any sensory experience or thought, since one cannot say “I think” or “I experience” without my already having a prior and implicit sense of I. Humphrey (2022) would have to disagree with Avicenna. On his view, the sense of self depends entirely on having sensory experience, which is equivalent to sentience and phenomenal consciousness. Take away sensory content and no self-awareness is possible.

The disagreement between Humphrey and Avicenna is framed in terms of content. In contrast, Harry Frankfurt suggests an answer that appeals to structure, and abstracts away from content:

What would it be like to be conscious of something without being aware of this consciousness? It would mean having an experience with no awareness whatever of its occurrence. This would be, precisely, a case of unconscious experience (Frankfurt, 1988, 162).

This is consistent with the phenomenological view, which suggests the positive formulation: if the super flying man were still conscious, he would necessarily be minimally self-aware since pre-reflective self-awareness is intrinsic to (or is part of the structure of) consciousness. Strawson and Zahavi, even in presenting an abstract phenomenology of the minimal self-experience, nonetheless hold that the phenomenon is real in the sense that there is in fact some irreducible experience of what it is like *for-me* in the very structure of every experience, whether that experience is complexly rich with sensory input or simple and impoverished in this regard. What it is like is always what it is like *for* someone. For phenomenologists like Husserl and Zahavi, this self-experience would hinge on the intrinsic temporal structure of consciousness. If this intrinsic temporality is a necessary and constituting component of minimal self-awareness, however, would it be sufficient, or would it even work, without sensory input of some sort?^15^

A less abstract and more embodied/enactive view is that both structure and content are important. Avicenna had been arguing against this view, especially as it was expressed in Aristotle, who suggests that what we call mind is not any real thing before it thinks or experiences (*De anima* 3.4, 429a23-24). That is, the mind and its structural features are enacted in the process of experiencing. Enactive views reflect this kind of self-production, often conceived as an autopoietic self-organizing process that involves a dynamical coupling of interoceptive, proprioceptive, and exteroceptive factors. Human experience is always complex—embodied and socially contextualized—but it also, arguably, always involves a minimal self-awareness. Avicenna may be right, however, that typically in one’s everyday life one does not know this minimal experience as such. One can gain insight into it only by engaging in certain practices—phenomenology, meditation, philosophical thought experiments, scientific experiments such as sensory deprivation experiments, and so on, all of which involve some degree of abstraction.

## Data availability statement

The raw data supporting the conclusions of this article will be made available by the authors, without undue reservation.

## Author contributions

SG: Writing – original draft, Writing – review and editing.

## Footnotes

1 Avicenna didn’t use the term “flying man.” Black (2008, 63 n. 3) attributes it to Gilson (1929-1930, 41 n. 1). The term “floating man” is also found in the literature.

2 Hasse (2000, 83) and Adamson and Benevich (2018) argue that in this context it means “essence,” rather than “self,” and contend that Avicenna is attempting to show in opposition to Aristotle that the essence of the soul does not include the body. In contrast, Kaukua (2015, 2020) maintains that the flying man argument was designed to point our attention to our being aware of ourselves independently of any other content of experience. For our purposes, we can follow Avicenna: “We say: what is intended by ‘the soul’ is that which each of us refers to by his saying, ‘I”’ (Avicenna (Ibn Sina), 1952, 183; trans. Marmura, 1986, 384). It is notably that G.E.M. Anscombe, without mentioning Avicenna, and rather focused on Augustine and Descartes, dreams up a very similar thought experiment about sensory deprivation to test whether it is the body to which each of us refers by saying, “I.” “Sight is cut off, and I am locally anaesthetized everywhere, perhaps floated in a tank of tepid water; I am unable … to touch any part of my body with any other” (Anscombe, 1975, 57).

3 Avicenna here is 180° removed from the view of Hume (1739/1978), who equates what we call “self” with the perceptions (sensations) we experience in introspection, and suggests “When my perceptions are remov’d for any time, as by sound sleep, so long am I insensible of myself, and may truly be said not to exist.” Also see Lane (2020) for considerations of whether the minimal self can dissociate from consciousness.

4 “Self-awareness is essential to the soul, it is not acquired from outside. It is as if, when the self comes to be, awareness comes to be along with it. Nor are we aware of [the self] through an instrument, but rather, we are aware of it through itself and from itself. And our awareness is an awareness without qualification, that is, there is no condition for it in any way; and it is always aware, not at one time and not another…. Self-awareness is natural (*gharîzah*) to the self, for it is its existence itself, so there is no need of anything external by which we perceive the self. Rather, the self is that by which we perceive the self.” (Avicenna (Ibn Sina), 1973, 160–162; trans. Black, 2008).

5 Strawson does not use the term “minimal self” in his 1997 and 1999 essays, but he does refer to the “minimal case” or form of self-experience. I may be to blame for the term “minimal self” in this phenomenological context (Gallagher, 2000a). I was referring specifically to Strawson’s account, and distinguishing minimal from narrative self. In that article I cite Damasio’s (1999) use of the term “core self” as a related concept. I also use the phrase in Gallagher (2000b), a volume edited by Dan Zahavi.

6 Strawson’s, 1997 paper generated four special issues in the *Journal of Consciousness Studies*, which I edited with Jonathan Shear, and which was then published as a volume, *Models of the Self* (Gallagher and Shear, 1999). Strawson (1999) provided a response to all of the essays. In this response he characterizes the minimal self as: [1] A subject of experience. [2] A thing, in some interestingly robust sense. [3] A mental thing, in some sense. [4] Single at any given time, and during any hiatus-free or strongly experientially unified period of experience. (1999, 108).

7 This is a point that runs throughout Hutto and Ilundáin-Agurruza’s (2020) essay in which they criticize the phenomenological concept of minimal self—namely the insistence that the minimal self is not something that we experience “as” self or *qua* self. “To acquire a sense of oneself *as a self* that is distinct from another—a sense of self such that one recognizes oneself *qua* self as featuring in shared experiencing—is a quite sophisticated conceptual achievement” (p. 518). Neither Strawson nor the phenomenologists characterize the minimal self in this way, however. Nor does Avicenna since he contends that we are not “alert” to this awareness, i.e., that we do not have reflective knowledge of it such that we take it *as* a self (1959, 226–227).

8 This is precisely the view expressed by the classic phenomenologists (Husserl, Sartre, Merleau-Ponty). That is, the notion of the minimal self is tied specifically to a subject’s pre-reflective self-awareness, and this kind of self-awareness is a structural feature of consciousness. The claim is that whenever I am conscious, I am pre-reflectively conscious of being conscious, that is, I am pre-reflectively aware that I am experiencing something. I have a self-awareness of my experience that does not depend on an additional act of consciousness that would reflectively take the first-order consciousness as an object.

9 There is not universal agreement about the connection between proprioception and the sense of body ownership, although in the rubber hand illusion, where one gains a sense of ownership for the rubber hand (it starts to feel as part of one’s body) the manipulation of proprioception is involved, so that it is subordinated to visual and tactile senses (see Limanowski, 2014). Also, in the absence of proprioception (as in cases of deafferentation) one’s body or body parts can feel alien (unowned) (Gallagher and Cole, 1995). Despite this and other evidence, Humphrey suggests that proprioception “is of little or no importance to establishing your sense of self” (2022, 133). It may be that he thinks of proprioception as purely a matter of physiological information and would reject the notion of proprioceptive awareness (see, e.g., Bermúdez et al., 1995 for this distinction)—according to Humphrey there is no phenomenal experience connected with proprioception (2022, 131).

10 For Husserl, we can come to this realization by means of a phenomenological reduction that sets aside any questions about causality. This just is the way that we experience things; and whether such experiences have a causal explanation in terms of neural, proprioceptive, or interoceptive processes is a different question. In this sense, the flying man argument effects something like a phenomenological reduction. If we could put ourselves in the situation of the flying man, we would have this type of pure phenomenological access to our experience.

11 One reviewer raised an important question about phantom limbs or a phantom body. Would a brain without any somatosensory or other bodily sensory input develop a sense of phantom bodily awareness? Even in cases of congenital absence of limbs individuals experience (aplasic) phantoms (Brugger et al., 2000; Brugger, 2011). One might assume that even the flying man, who, rather than being born, arrives fully mature but without bodily senses, might experience a phantom body. The issue is complicated. A traditional view, which denied aplasic phantoms, maintained that having a phantom depended on having had sensory experience with the relevant limb (e.g., Simmel, 1961). The current neuroscientific view is that somatosensory areas of the brain that would be correlated to the missing limb, even if they deteriorate without sensory input, may still generate a phantom. What would Avicenna think? The first mention of phantoms has been attributed to Ambroise Paré in the 16th century. But even if, as Björn Meyerson (in Finger and Hustwit, 2003) suggests, Avicenna in his medical practice must have encountered the phenomenon of phantom pain, it’s not clear how he would go about explaining it. Clearly, we should not attribute an understanding of contemporary neuroscience, plasticity or neural reorganization to him. We could ask what the flying man’s brain would be like. Since Avicenna indicates that he is “created all at once, and created as perfect,” we would expect that he came into existence with a perfectly normal brain but in a complete sensory deprivation condition. In this condition would he experience a phantom body (or body part)? What stimulus would spark this experience of a phantom. If we think that some sensory experience or motor reafference is required, these, as well as bodily pain, phantom or not, are supposedly ruled out by the experiment. And if the phantom was generated by a completely spontaneous activation of the somatosensory cortex, for example, then from the perspective of the flying man’s experience this would be the equivalent of a dream-like phantom or illusion. The question about phantoms is an interesting one, but not one that is easily answered. In this respect Avicenna states: “If in this situation [of the flying man] he were able to imagine a hand or another limb, he would not imagine it as a part of himself, nor as a condition for his self….” The same might be said in regard to the hallucinations that sometimes occur in sensory deprivation experiments (Vosburg et al., 1960; Mason and Brady, 2009).

12 The vestibular sense is another complication and is connected with the idea that the flying or floating man could still have a bodily sense of floating of flying or hovering (as may occur in experiments on out-of-body experiences—Blanke, 2004). Indeed, the vestibular sense may be increased with the loss of other sensory inputs (Horak and Hlavacka, 2001). It’s difficult to discuss vestibular sense on its own since it is tightly connected with other senses (including vision and somatosensory input), and without the other senses it’s difficult to know how the vestibular sense would function. It’s also the case that one can lose vestibular sense, so, again following Avicenna’s aim, we can stipulate the elimination of the vestibular sense.

13 Even if the phenomenology of sensory deprivation came close to the flying man situation (which it doesn’t for reasons stated above), typically the subjects of such experiments are not newly created perfect humans. Also, disruptions of interoception are often associated with experiences of dissociation (e.g., Pick et al., 2020; Kaldewaij et al., 2023).

14 As one reviewer suggested, the super flying man may just be what Avicenna intended as the flying man.

15 For the phenomenologists the answer is not clear cut. It depends on how one conceives of the relation between intrinsic temporality, intentional structure, and sensory content (which Husserl calls “hyletic” content), and at least on one embodied interpretation these features of consciousness mutually constrain each other (see e.g., Williford, 2013; Zippel, 2014; Soueltzis, 2023).
